# In Silico and In Vitro Methods in the Characterization of Beta-Carotene as Pharmaceutical Material via Acetylcholine Esterase Inhibitory Actions

**DOI:** 10.3390/molecules28114358

**Published:** 2023-05-26

**Authors:** Arunachalam Muthuraman, Muthusamy Ramesh, Fazlina Mustaffa, Ahmed Nadeem, Shamama Nishat, Nallupillai Paramakrishnan, Khian Giap Lim

**Affiliations:** 1 Pharmacology Unit, Faculty of Pharmacy, AIMST University, Bedong 08100, Malaysialim.khian.giap@gmail.com (K.G.L.); 2 Department of Pharmaceutical Analysis, Omega College of Pharmacy, Hyderabad 501301, India; shreeramesh@gmail.com; 3 Department of Pharmacology and Toxicology, College of Pharmacy, King Saud University, Riyadh 11451, Saudi Arabia; anadeem@ksu.edu.sa; 4 Comprehensive Cancer Center, Wexner Medical Centre, Ohio State University, Columbus, OH 43210, USA; shamama.nishat@gmail.com; 5 Department of Pharmacognosy, JSS College of Pharmacy, Mysore, JSS Academy of Higher Education and Research, Mysore 570015, India

**Keywords:** Alzheimer’s disease, acid phosphatase, galatamine, neurovascular disorders, rivastigmine, vascular dementia, zebrafish embryo toxicity test

## Abstract

Molecular docking is widely used in the assessment of the therapeutic potential of pharmaceutical agents. The binding properties of beta-carotene (BC) to acetylcholine esterase (AChE) proteins were characterized using the molecular docking method. The mechanism of AChE inhibition was assessed by an experimental in vitro kinetic study. In addition, the role of BC action was tested by the zebrafish embryo toxicity test (ZFET). The results of the docking ability of BC to AChE showed significant ligand binding mode. The kinetic parameter, i.e., the low AICc value shown as the compound was the competitive type of inhibition of AChE. Further, BC also showed mild toxicity at a higher dose (2200 mg/L) in ZFET assessment with changes in biomarkers. The LC_50_ value of BC is 1811.94 mg/L. Acetylcholine esterase (AChE) plays a pivotal role in the hydrolysis of acetylcholine, which leads to the development of cognitive dysfunction. BC possesses the regulation of acetylcholine esterase (AChE) and acid phosphatase (AP) activity to prevent neurovascular dysfunction. Therefore, the characterization of BC could be used as a pharmaceutical agent for the treatment of cholinergic neurotoxicity-associated neurovascular disorders such as developmental toxicity, vascular dementia, and Alzheimer’s disease due to its AChE and AP inhibitory actions.

## 1. Introduction

Dementia is one of the types of neurovascular dementia. Alzheimer’s disease (AD) and vascular dementia (VaD) are very common types of neurocognitive disorders. AD is a neurodegenerative disease of the central nervous system. The progression of AD leads to dementia, behavioral abnormalities, and cognitive impairments [1] with alteration in cholinergic neurotransmission. AD and VaD are a type of progressive neurodegenerative disorders that is the most common cause of dementia among the elderly individuals, affecting over 50 million people worldwide according to a 2019 estimate by ADI, among other neurological conditions [2]. Individuals with AD and VaD exhibit neuronal cell death via alteration acetylcholine homeostasis, amyloid peptide depositions in nerve and blood vessels, formation of neuritic plaques, and neurofibrillary tangles in the nerve cells of the brain [3]. Cholinergic function in the body and brain is primarily mediated by acetylcholine, which is the most prevalent neurotransmitter in the body. The excessive level of acetylcholinesterase (AChE) hydrolyses the acetylcholine neurotransmitter. According to the “cholinergic hypothesis”, the mechanism of neurodegeneration occurs due to the reduction in acetylcholine levels in the brain [4]. There is a vast amount of evidence indicating that inhibitors of AChE can impede the progression of AD and VaD. The drugs currently utilized to treat AD and VaD patients function by increasing the level of acetylcholine in the brain via activation of central cholinergic biosynthesis and neurotransmission.

The administration of AChE inhibitors prevents the hydrolysis of acetylcholine and regulates neurocognitive function. However, the existing AChE inhibitor shows unwanted side effects such as hepatotoxicity, urinary incontinence, bradycardia, pulmonary disorders, and unintended weight loss [3]. Therefore, AChE-targeted inhibitors with more effective and safer pharmaceutical agents are still needed for better treatment of neurocognitive disorders via regulating cholinergic neurotransmission. The various medicinal plants and their constituents (Palm-Oil-Derived BC, rutin, alpha-napthoflavone, and curcumin) were proven to ameliorate neurovascular disorders via AChE inhibitory actions without toxic effects due to their multitargeted actions and antioxidant potentials [5,6,7,8,9,10]. Experimentally, tocotrienol-rich fraction and BC possess the ameliorative effect against the streptozotocin-induced diabetic VaD in rats via AChE inhibitory actions [5,6,11,12]. However, the biophase effect and metabolic intermediate may be possible for this in vivo pharmacological AChE inhibition. The current research was focused on exploring the direct effect of BC on AChE inhibitory action via molecular docking and in vitro kinetic assessment. Furthermore, the ZFET is a more reliable, robust, and rapid method of assessment of the toxicity of the pharmaceutical agent. The novel AChE inhibitors from natural sources are expected to produce better efficacy and be safer for the management of neurodegenerative disorders such as AD and VaD. Furthermore, the additional detoxification enzyme, i.e., acid phosphatases (AP) is also involved in the regulation of neurovascular functions [13]. The metabolic hydrolysis of acetylcholine and phosphomonoesters are catalyzed by AChE and AP enzymes, respectively, and contribute to the developmental process of the embryo [14,15,16]. The current study was intended to prove the direct inhibition of the effect of BC on AChE activity via the ZFET method. Hence, the present study is designed to produce more promising evidence of BC action on the direct inhibition of AChE activity via in silico and in vitro studies.

## 2. Results

### 2.1. Ligand Binding Landscape Analysis of BC

The docked complex of BC, rivastigmine, and galantamine exhibited docking scores ranging from 27.7 to −7.3 kcal/mol. BC displayed a binding mode with acetylcholine esterase, as indicated by a docking score of 27.7 kcal/mol and 14.1 kcal/mol, respectively, into the crystal structures of 1GQR and 1QTI. The binding mode of BC tends to occupy the binding site of the reference ligands although the binding affinity is low in comparison to the reference ligands. Trp81, Gly114, Gly115, Gly116, Val126, Glu196, Trp276, Leu279, Asp282, Ser283, Phe328, Gly438, and Ile441 are the important amino acids that have been found in the crystal structure of PDB ID: 1GQR that may interact with BC within the 5 Å region. Val68, Asp69, Glu70, Gly114, Gly115, Glu196, Trp276, and Gly438 are critical amino acids surrounding BC in the crystal structure of PDB ID: 1QTI. The molecular docking scores of ligands with the crystal structures of acetylcholine esterase and its important binding site residues are listed in Table 1.

**Figure 1:**
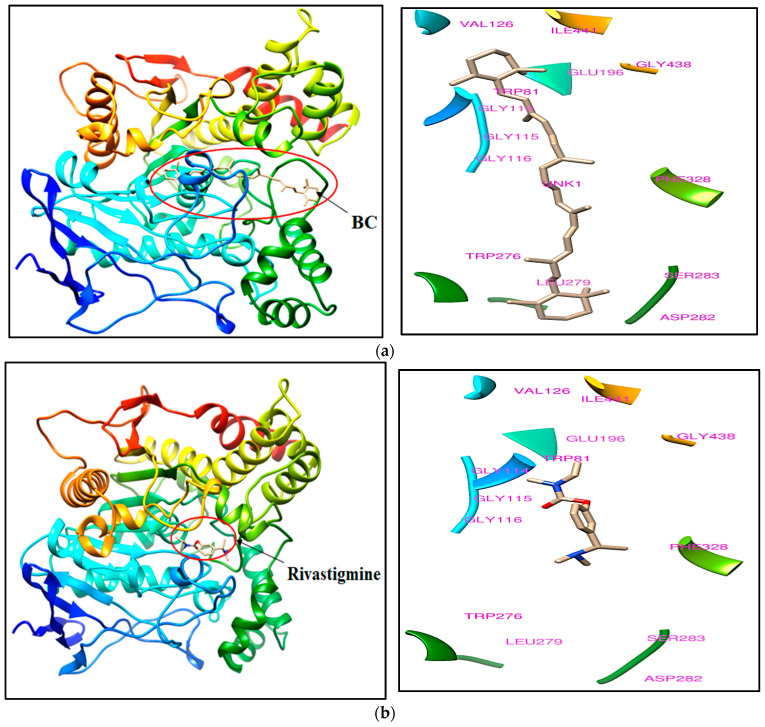
The docking pose of (**a**) BC and (**b**) rivastigmine (bound ligand) within the crystal structure of acetylcholine esterase (PDB ID: 1GQR). Abbreviation: BC, beta-carotene; PDB-ID, protein data bank ID.

**Figure 2:**
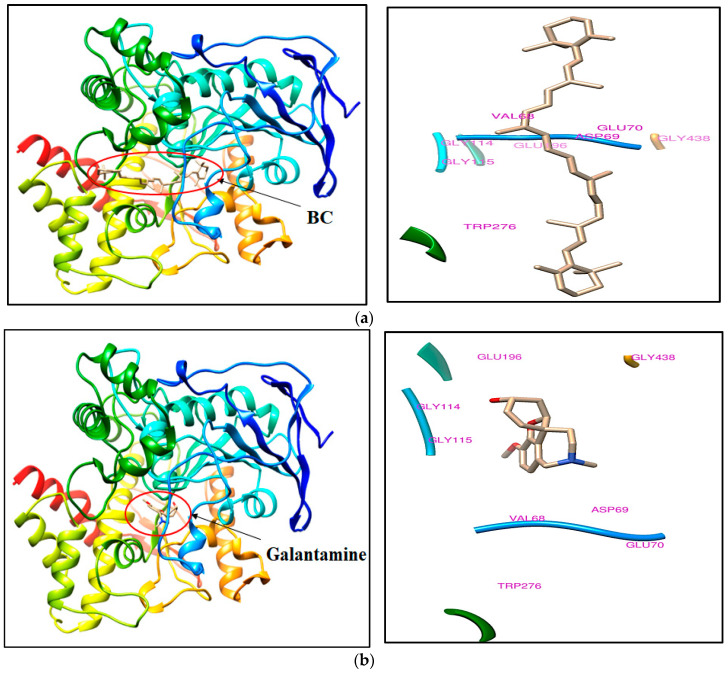
The docking pose of (**a**) BC and (**b**) galantamine (bound ligand) with the crystal structure of acetylcholine esterase (PDB ID: 1QTI). Abbreviation: BC, beta-carotene; PDB-ID, protein data bank ID.

BC has been found to bind at the active site, although docking scores are low in comparison to the bound ligands. The bound ligands such as rivastigmine and galanthamine have shown tight binding affinity at the binding site of acetylcholine esterase with the values of, respectively, −7.3 and −9.8. The docking pose of BC and rivastigmine with the crystal structure of acetylcholine esterase (PDB ID: 1GQR) is illustrated in Figure 1a,b, respectively. Similarly, the docking pose of BC and galantamine with another crystal structure of acetylcholine esterase (PDB ID: 1QTI) is illustrated in Figure 2a,b, respectively.

### 2.2. In Vitro Kinetic Assessment of Acetylcholine Esterase Inhibition

Kinetic studies of the AChE enzyme inhibitor compound were analyzed to interpret the inhibition mechanism of the compound. The mechanism of enzyme inhibition was analyzed by graph fitting analysis using the Sigma-Plot enzyme kinetic software (Systat Software v.1.10 Inc., San Jose, CA, USA) By using various kinetics plots such as the Lineweaver-Burk, Hill, Hanes-Woolf, Eadie-Hofstee, Dixon, and Scatchard models, the kinetic parameters such as maximum velocity (Vmax), Michaelis constant (Km), Akaike information criterion (AICc), and significant correlation variable (R^2^) values of BC and donepezil were calculated. In comparison, the in vitro kinetic assessment of BC and donepezil on acetylcholine esterase inhibition showed potential actions. The details of the kinetic parameters of BC and donepezil for the inhibition of acetylcholine esterase activity are presented in Table 2.

**Figure 3:**
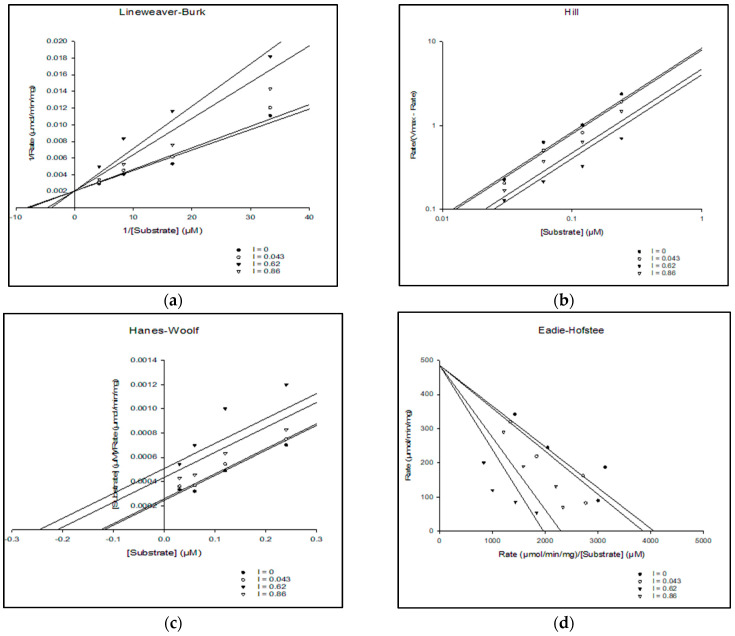

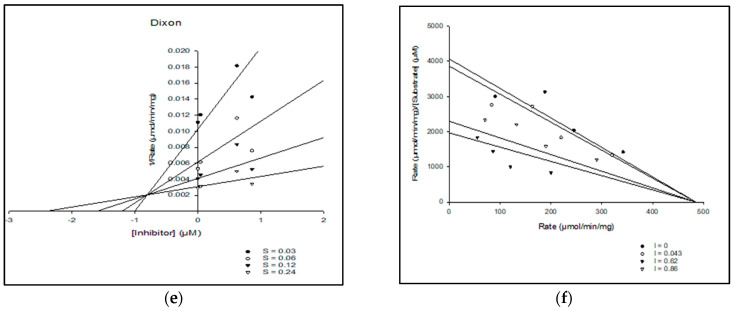
The assessment of acetylcholine esterase inhibition of BC by kinetic studies. (**a**) Lineweaver–Burk plot of 1/[S] vs. 1/Rate in the different concentrations of competitive-type inhibitor. (**b**) Hill plot of different concentrations of [S] vs. Rate (Vmax Rate) in the different concentrations of competitive-type inhibitor. (**c**) Hanes–Woolf plot of the compound at different concentrations of [S] vs. [S]/Rate in the different concentrations of competitive-type inhibitor. (**d**) Eadie–Hofstee plot of the compound by rate/[S] vs. Rate in the different concentrations of competitive-type inhibitor. (**e**) Dixon plot of the compound at different concentrations of competitive inhibitor vs. 1/Rate. (**f**) Scatard plot of the compound by Rate vs. Rate/[S] in the different concentrations of competitive-type inhibitor.

Based on the curve fitting model and the low AICc value, the kinetic study results show that the compound follows a competitive-type inhibitor mechanism. The assessment of the acetylcholine esterase inhibition of BC by kinetic studies is illustrated in Figure 3.

### 2.3. Effect of BC in ZFET

**Figure 4:**
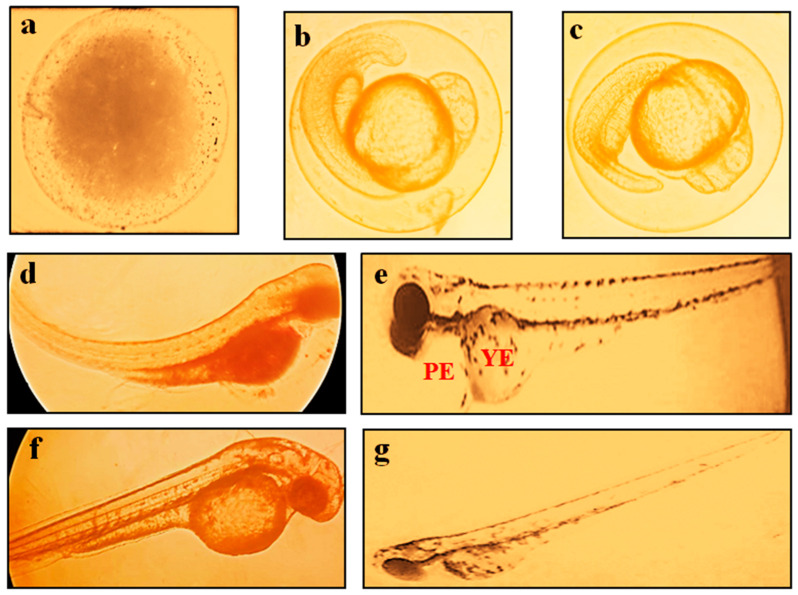
State of embryo development with exposure to BC in ZFET. (**a**) shows the coagulated embryo. (**b**,**c**) shows the development abnormalities, i.e., lack of tail coil detachment and dechorionation of the zebrafish embryo. (**d**) shows the spondylitis. (**e**) shows the development of PE and YE. (**f**) shows the eye (size reduction) deformation and abnormalities of the heartbeat. (**g**) shows the complete survival of the embryo without malformations. Abbreviations: PE, pericardial edema; and YE, yolk edema. The observations were made with a stereomicroscope under 40× magnification.

The exposure of the E3 medium control group showed the healthy development of the embryo and the release of larvae without any malformation. The exposure of BC (2200 mg/L) and DMSO showed the malformation of the embryo (i.e., coagulation and lack of dechorionation) and the larvae development of the body, spondylitis with development of pericardial edema (PE), yolk edema (YE), eye deformation (small eyes), and abnormalities of a heartbeat. The state of embryo development with exposure to HFE-ME in ZFET is shown in Figure 4.

**Figure 5:**
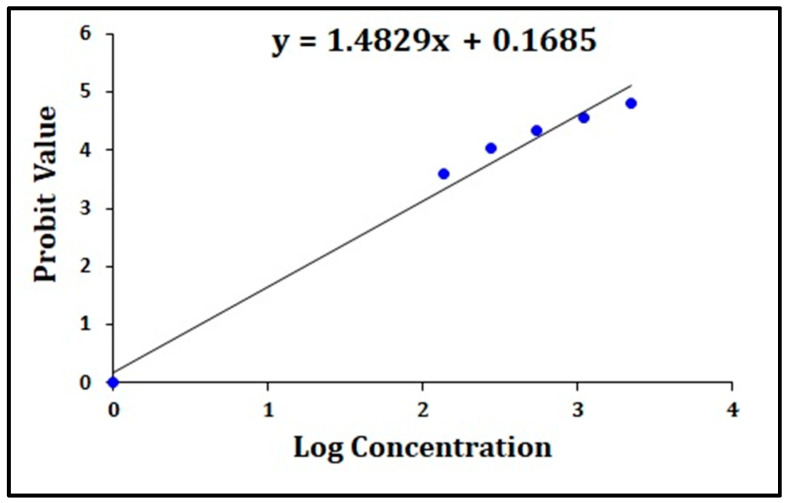
The log concentration–probit linearity graph.

Based on this observation, the value of BC concentration and % mortality were converted to log concentration and probit values, respectively, by using Finney’s Probit analysis table chart [17]. The linear formula was obtained with log concentration (*x*-axis) versus probit values (*y*-axis) in an Excel graph. The log concentration–probit linearity graph and % mortality with the corresponding BC concentration are illustrated in Figure 5.

Calculation: The x-value of the linear formula was representing the LC_50_ log. This log LC_50_ value was further converted to a normal value for obtaining the LC_50_ value of the HFE-ME exposure in the zebrafish embryo. The calculation steps are as follows:

y = 1.4829 x + 0.1685,

x (Log LC_50_) = (5 − 0.169)/1.483 = 3.258,

LC_50_ = 1811.94.

Hence, the LC_50_ value of zebrafish embryos with BC exposure was 1811.94 mg/L.

### 2.4. Effect of BC in AChE and AP Activity Levels in the Zebrafish Embryo

The exposure of BC (2200 mg/L/day for 6 days), DMSO (10% for 6 days) and donepezil (5 ng/mL for 6 days) showed statistically significant (*p* < 0.05) rising of tissue AP (EC 3.1.3.2) activity levels as an indication of developmental toxicity when compared to embryo medium (E3) exposure. There are no changes in AP activity at the dose of 137.5, 275, 550, and 1100 mg/L/day of BC in the 6 days of exposure. Meanwhile, the donepezil (5 ng/mL for 6 days) treatment showed a significant difference in AChE and AP activity when compared to the DMSO control group. The results of the AP activity are presented in Table 3.

## 3. Discussion

The molecular docking study revealed that BC possesses the ability to dock to AChE proteins (PDB ID: 1GQR and 1QTI) with the scores of 27.7 kcal/mol and 14.1 kcal/mol in respective PDB AChE proteins. Although the docking scores are low, the resulting binding mode of BC was similar to rivastigmine and galantamine, which indicates that the BC possess strong binding landscape properties with brain AChE protein. The in vitro kinetic studies of AChE enzyme inhibitor by graph fitting analysis, i.e., the Lineweaver-Burk, Hill, Hanes-Woolf, Eadie-Hofstee, Dixon, and Scatchard models show the changes in maximum velocity (Vmax), Michaelis constant (Km), Akaike information criterion (AICc), and significant correlation variable (R^2^) values for the inhibition of AChE. The in vitro 144 h zebrafish embryo toxicity test (ZFET) showed a mild effect on zebrafish embryos, i.e., the coagulation embryo; developmental abnormalities such as the lack of tail coil detachment and dechorionation of the zebrafish embryo; spondylitis; pericardial edema (PE); yolk edema (YE), eye deformities; and abnormalities of the heartbeat. Furthermore, the calculated value of the LC_50_ dose of BC in ZFET was 1811.94 mg/L. It indicates that BC has the least toxicity in morphometric analysis of the zebrafish embryo development. However, the higher concentration of BC (2200 mg/L) makes changes in biomarker, i.e., AP activity, and interferes with the development of the embryo at various stages.

The controlled action of detoxification enzymes, i.e., AChE and AP are employed in the potential role in the development of embryos [18]. The elevated levels of AChE and AP alter the cellular function via the reduction in acetylcholine and phosphate esters [18,19]. These products and their chemical reaction are essential for different metabolic processes in embryos, skeletal muscle growth, as well as neurovascular functions [20,21,22]. The AP activity modulates the embryonic development of chicken eggs [23]. The raising of AP is accompanied by a reduction in plasma ChE activity with chronic exposure to organophosphates in hens. This relationship is also documented to reduce and delay the neurotoxic effect of organophosphate esters [24]. Furthermore, the fertilization of oocytes is triggered by acid phosphatase activity during the embryogenesis of *Rhodnius prolixus* [20] and *Drosophila melanogaster* [25]. Carotenoids such as astaxanthin and BC enhance the marked tolerance against hydrogen peroxide-induced mortality, oxidative stress, and apoptosis which leads to an improved egg quality and acts as a nutrient source [26]. Similar results are also obtained in this research work. In contrast, BC interferes with the lipoprotein metabolism process in the maternal–fetal barrier and expression of genes for organogenesis due to the active accelerated metabolic process of BC by the developing tissues [27]. A higher dose of the readily stored BC and the rapid conversion of BC to vitamin A leads to changes in the retinoid receptor-mediated abnormal expression of mitochondrial genes, which, in turn, leads to alteration if various barrier functions, malformations, and lethality of the embryo [28,29].

However, an extreme level of BC intake can cause changes in skin tone along with itching, abdominal cramps, exhaustion, and weight loss. Indeed, the higher dose of β-carotene supplementation also enhances hepatic damage in alcoholic patients [30,31,32]. The pathological mechanism of BC is converting it to vitamin A, which leads to abundant accumulation of vitamin A stored in hepatic stellate cells, causing cellular hypertrophy, excessive biosynthesis of collagen, fibrosis, and hepatic damage [33]. The major limitation of this outcome focuses on the cholinergic neurotransmission-associated functions [6]. However, BC plays a protective role in lipoprotein metabolism at various levels including the maternal–fetal barrier conditions [27]. Moreover, BC supplements raise the risk of developing lung cancer in smokers [34,35]. Similarly, the higher dose of BC diminishes the cardioprotective action against the ischemia/reperfusion-induced oxidative stress in rats [36]. Hence, BC is a friend (anti-oxidant) or foe (pro-oxidant) for the therapeutic actions of various neurovascular disorders, and their mechanisms remain elusive. Therefore, more extensive studies of BC and vitamin A (retinol) action in various tissue protections need to be conducted with suitable markers to explore pathological mechanisms in various conditions in experimental animal models and clinical trials.

## 4. Materials and Methods

### 4.1. In Silico Computational Studies to Characterize the Ligand Binding Mode

The process of molecular docking is a computational technique that can be used to identify the ligand binding mode and the interaction between a ligand and a protein. In this particular study, BC was subjected to molecular docking to predict its affinity towards acetylcholine esterase. The recently reported crystal structure of acetylcholine esterase (PDB ID: 1GQR and PDB ID: 1QTI) was used to conduct the molecular docking process [37]. Autodock vina integrated with the Chimera software package was utilized to carry out the molecular docking process with the pre-constructed and energy-minimized ligands (BC, rivastigmine, and galanthamine) [38]. The 3D structure of the minimized ligands was further prepared for ionization state, charge, etc. The crystal structure of acetylcholine esterase was also prepared in the Chimera software by fixing the structure with the missing hydrogen and removing water molecules. The ligands were docked at a 25 Å sphere that was generated around the bound ligand region of acetylcholine esterase. The results of molecular docking were then analyzed based on the docking score and binding pose to estimate the ligand–protein interactions and binding landscape.

### 4.2. In Vitro Kinetic Studies to Determine the ACE Inhibition Mechanism of the Ligand

To determine the mechanism of AChE enzyme inhibition, the kinetic studies were performed using Ellmans Methods and varying concentrations of inhibitors (0, 0.43, 0.86 µM) with different concentrations of the inhibitor substrate (butyrylthiocholine iodide (BTCI)) including 0.03, 0.06, 0.12 and 0.24 µM. The enzyme was dissolved in de-ionized water to produce the concentration of 0.2 mg/mL for each well, and the inhibitor substrate was dissolved in a sodium phosphate buffer. The reaction mixture was incubated for 30 min at 25 °C in 1 min intervals. The absorbance was measured at 412 nm using an ELISA reader by keeping the 96-well plate (BioTek XL-800, Taunton, MA, USA). The kinetic parameters Vmax, Km, Ki, AICc, and R^2^ values of the AChE Inhibitor were determined by graph fitting analysis using the Sigma-Plot enzyme kinetic software 14.0 version [39,40].

### 4.3. Study of BC in Zebrafish Embryo Toxicity Test (ZFET)

Zebrafish embryos were collected by using a specialized breeding tank as described by Adatto et al. [41] with a slight modification of Gonsar et al. [42]. Briefly, the tank was constructed with slanting curved nylon mesh inside of the rectangular 5L breeding tank. Both male and female (2:1 ratio) wild type of adult zebrafishes were placed in this breeding tank and allowed to mate from one day before (17:00 h) to the next morning (9:00 a.m.). During this period, tank aerators were applied, and this setup was kept in the laboratory glass door for the natural morning light to pass through the tank. At 10:00 a.m., the fish were taken out and placed in the home tank. Then, the mesh was removed from the tank, and the fertilized eggs were collected from the bottom of the breeding tank. The fertilized eggs were observed under a stereomicroscope at a 4× magnification. Non-fertilized eggs were removed from the acute toxicity study. The 96-well microplates (flat bottom; 400 µL) were used for acute toxicity study. Each well carried one fertilized egg. The embryos were kept in 200 µL of embryo medium (E3). The E3 medium was prepared by mixing a 60× stock solution. The stock solution was prepared by dissolving 34.8 g of sodium chloride (NaCl); 1.6 g of potassium chloride (KCl); 5.8 g of calcium chloride (CaCl_2_·2H_2_O); and 9.78 g of magnesium chloride (MgCl_2_·6H_2_O) in 1.95 L of double distilled water. The pH of the E3 medium was adjusted to 7.2 using sodium hydroxide (NaOH) and hydrochloric acid (HCl). Crucially, the final volume was adjusted to 2 L with double distilled water. Then, 200 µL of extracts with variable concentration (geometric series) were filled in each well as per the guidelines of the Organization for Economic Co-operation and Development (OECD 236: FET, fish embryo toxicity test) [43]. The microplate was kept at 28 °C throughout the acute toxicity study.

**Figure 6:**
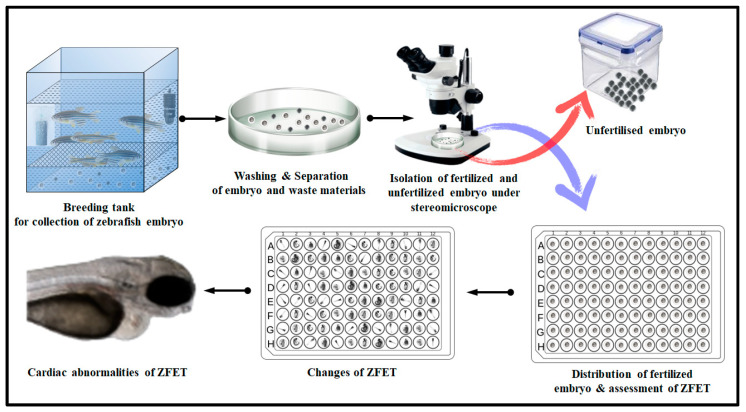
The procedure for ZFET. Abbreviation: ZFET, zebrafish embryo toxicity test.

The detailed experimental protocol group distributions are as follows: Group 1: Naïve control group was exposed to only E3 medium. Group 2: This group was exposed to a BC dose of 137.5 mg/L for 24 h for 6 days. Group 3: This group was exposed to a BC dose of 275 mg/L for 24 h for 6 days. Group 4: This group was exposed to a BC dose of 550 mg/L for 24 h for 6 days. Group 5: This group was exposed to a BC dose of 1100 mg/L for 24 h for 6 days. Group 6: This group was exposed to a BC dose of 2200 mg/L for 24 h for 6 days. Group 7: This group was exposed to a 10% dimethyl sulfoxide (DMSO) for 24 h for 6 days. Group 8: This group was exposed to a 10% donepezil (5 ng/mL) for 24 h for 6 days. Each group consisted of twelve embryos. The study was performed three times to obtain reliable reproducible data. This experimental protocol was duly approved by the AIMST University Animal Ethics Committee of AIMST University (AUAEC/FOP/2023/12). The summarized procedure for ZFET is illustrated in Figure 6.

The calculation of the 50 percent lethal concentration (LC_50_) of zebrafish embryo toxicity was performed by the arithmetic Karber method [44] and Finney’s Probit analysis method [17]. Briefly, the concentration was converted to a log concentration, and % mortality converted to probit values was converted using Finney’s table. Thereafter, the graph was designed with the log concentration (*x*-axis) versus the probit values (*y*-axis) in Excel to obtain the linear formula. Here, the x-value of the linear formula was known as log LC_50_, and the calculated log LC_50_ value was converted to LC_50_ using the anti-logarithmic method.

### 4.4. Estimation of AP Activity

The AP activity was estimated as described by Neil and Horner [45]. Briefly, the embryo was euthanized by hypothermic shock (containing 5 parts of ice and 1 part of system water) for 40 min. Then, each group of embryos was homogenized (10% *w*/*v*) in 10 mL of ice-cold 50 mM citrate buffer at pH 5.3 with ultra-cooled mortar and pestle. Further, homogenates were filtered with multilayered cheesecloth. Then, the filtrate was centrifuged at 10,000× *g* for 10 min. The 0.5 mL of clear aliquot was mixed with 3 mL of substrate solution. The substrate solution was prepared by dissolving 1.49 g of ethylenediaminetetraacetic acid, 0.84 g of citric acid, and 0.03 g of *p*-nitro-phenyl phosphate in 100 mL of distilled water. The pH was adjusted to 5.3. The prepared mix was incubated for 5 min at room temperature (37 °C). The 0.05 mL sample was removed immediately from the above solution and mixed with 9.5 mL of sodium hydroxide (0.085 N). The principle of AP activity is to hydrolyze the *p*-nitro-phenol phosphate substrate and produce the yellow color chromogen *p*-nitro-phenol in an alkaline condition. The changes in color absorbance were measured at a wavelength of 405 nm using a spectrophotometer (Shimadzu Corporation, Kyoto, Japan). The 0–20 mM of *p*-nitro-phenol diluted with 10 mL of sodium hydroxide solution was used as the reference standard.

### 4.5. Estimation of AChE Activity

The AChE activity was estimated as described by Ellman [39]. Briefly, about 0.5 mL of tissue supernatant was mixed with 2.5 mL of phosphate-buffered saline (pH 7.4) and 0.1 mL of 5,5–dithio–bis–(2–nitrobenzoic acid) (DTNB, Ellman reagent) solution was added. The absorbance changes were noted using the Shimadzu UV–1800 UV/Visible Scanning Spectrophotometer (Shimadzu Corporation, Kyoto, Japan) at a wavelength of 412 nm. A total of 20 μL of acetylthiocholine iodide was added as a substrate. The changes in yellow color chromogen absorbance value were noted every 2 min until the absorbance value became constant. The AChE activity was calculated using the following formula:

$$Acetylcholinesterase\;activity=\frac{\Delta A}{13,600}+\frac{1}{\left(\frac{\mathrm{VBs}}{\mathrm{TVts}}\right)P},$$

where ∆A is a change in absorbance/min; P is protein content (mg/mL); VBs is the volume of brain supernatant (VBs = 500 µL); and TVts is the total volume of test samples (TVts = 3120 µL). The AChE activity was expressed as μM of acetylthiocholine hydrolyzed/mg of protein/minute.

### 4.6. Estimation of Tissue Total Proteins

The tissue total proteins were estimated as described by Lowry et al. [46]. Briefly, the aliquot and Lowry reagent mixture mixed well, and the change in absorbance of purple color chromogen was noted spectrophotometrically (DU 640B, UV-Spectrophotometer, Beckman Coulter Inc., Brea, CA, USA) at a 750 nm wavelength. The results were expressed as mg of protein per ml of the aliquot solution.

### 4.7. Statistical Analysis

Data were statistically analyzed by the non-linear regression model method to calculate the LC_50_ values of BC as described by Hedgpeth et al. [47]. Furthermore, the LC_50_ logistic regression model to fit the numerical calculations was confirmed by using graphical summaries with Probit analysis. Confidence intervals (95%) for the LC_50_ were likelihood compared with Busquet et al. [48]. The data of tissue biomarkers were analyzed by one-way analysis of variance (ANOVA) followed by post hoc analysis with Tukey’s honestly significant difference test. The alpha value was considered as >0.05. All calculations were performed using the Graph Pad Prism version 5.03 software.

## 5. Conclusions

The results obtained from in silico and in vitro studies for assessing AChE enzyme inhibition activity demonstrate that BC exhibited a remarkable competitive inhibitory effect. In addition, in vitro embryo toxicity test of BC showed inhibition of AP activity. These findings suggest that the BC can be used as natural pharmaceutical material for the treatment of cholinergic neurotoxicity-associated neurovascular disorders such as vascular dementia and Alzheimer’s disease due to its AChE and AP enzyme inhibitory actions.

## Figures and Tables

**Table 1:** Molecular docking scores of BC into the crystal structures of acetylcholinesterases.

No.	Ligands	Docking Scores	Important Binding Site Residues
**Acetylcholine Esterase (PDB ID:1GQR)**			
1	BC	27.7	Trp81, Gly114, Gly115, Gly116, Val126, Glu196, Trp276, Leu279, Asp282, Ser283, Phe328, Gly438 and Ile441
2	Rivastigmine (Bound ligand)	−7.3	
**Acetylcholine Esterase (PDB ID:1QTI)**			
1	BC	14.1	Val68, Asp69, Glu70, Gly114, Gly115, Glu196, Trp276, and Gly438
2	Galanthamine (Bound ligand)	−9.8	

Here, BC, beta-carotene; PDB-ID, protein data bank ID.

**Table 2:** The kinetic parameters of BC and donepezil for the inhibition of acetylcholine esterase activity.

Compounds	Vmax (µM/min)	Km (mM)	AICc	R^2^	Type of Inhibition
BC	642.9	0.19	160.2	0.986	Competitive type
Donepezil	107.8	0.18	85.2	0.921	Mixed type

Here, BC, beta-carotene; AICc, Akaike information criterion; Km, Michaelis constant; R^2^, significant correlation variable; and Vmax, maximum velocity.

**Table 3:** Effect of BC in the changes in embryonic tissue AChE and AP activity.

Groups	AChE Activity (μmol of AChI/min/mg of Protein)	AP Activity (mM)
Naïve (E3 medium)	0.21 ± 0.03	0.9 ± 0.02
BC (137.5)	0.23 ± 0.06	1.3 ± 0.01
BC (275)	0.25 ± 0.02	1.1 ± 0.03
BC (550)	0.20 ± 0.04	1.2 ± 0.02
BC (1100)	0.29 ± 0.08	1.4 ± 0.04
BC (2200)	0.52 ± 0.13 ^a^	1.8 ± 0.08 ^a^
DMSO (10%)	0.43 ± 0.08 ^a^	2.4 ± 0.12 ^a^
Donepezil (5 ng/mL)	0.13 ± 0.07 ^b^	1.1 ± 0.03 ^b^

The numbers in parentheses represent a dose of mg/L. The results are presented as the mean SEM, with *n* = 12 embryos per group. ^a^ *p* < 0.5 versus the naïve control group. ^b^ *p* < 0.5 versus the DMSO control group. Abbreviations: AP, acid phosphatase; BC, beta-carotene; and DMSO, dimethyl sulfoxide.

## Data Availability

Data available on request.

## Sample Availability

Samples of the compound is available from the corresponding author.
